# Diseases of the Joints

**Published:** 1887-06

**Authors:** 


*Diseases of the Joints.* By HOWARD MARSH, F.R.C.S. Pp. 461. London: CASSELL & Co. 1886.

This, the latest of Cassell's excellent series of Clinical Manuals, is scarcely to be reckoned among the best. Possibly it is not easy to maintain the high standard to which the first numbers in the series rose; still, a drop from high excellence to mediocrity in the progress of an undertaking is disappointing. Not only is this work not up to the standard of the series; it is distinctly inferior to at least two other well-known works in the English language on the same subject.

The work is in no sense original. This we should not complain of if the author had made himself familiar with the original work of others. The pathology is done by proxy, and is, naturally, somewhat scurvily treated. In these days we like to believe that the most scientific treatment is worked out from the most perfect pathology. In this work pathology and treatment are as the poles asunder; not only are they written by different men—they might have been written in different languages, and even in separate books. There is not a shadow of an attempt to harmonise them. We do not affirm that the pathology is not sound, but it is meagre or beside the mark, and it has not been welded into the structure of the work.

In the purely clinical descriptions the author is at his best. But here also something is to be desired. The author has confined himself too strictly to his own experience. Now, experience is the best of teachers, if it is active and exploring, and not passive merely: experiment as well as observation should contribute to it. The three hospitals with which Mr. Marsh is connected ought to have provided a sufficiently wide field for experiment: it is just conceivable that, the area being so wide, the surface only could be tilled. In a word, the author's world is St. Bartholomew's Hospital. He describes his own position himself (p. 320): "I feel assured, in expressing the opinion I have given above, by the knowledge that my views are in full accordance with those of my colleagues both at St. Bartholomew's Hospital and at the Hospital for Sick Children." Now, St. Bartholomew's Hospital is not the world, even with the Hospital for Sick Children thrown in. It is more than an act of grace, it is a duty, to pay some attention to the work of others outside one's own hospital.

The best parts of the work are those dealing with Epiphysitis, Internal Derangement, Bone-setting, and Nervous Mimicry; and the descriptions of special diseases of special joints are clear and trustworthy. The least satisfactory chapters are those dealing with scrofulous diseases of joints, and generally with inflammatory and suppurative disease. The manner in which he speaks of aspiration of over-distended joints made us rub our startled eyes and wonder if in this matter also his colleagues are with him. It is clear that the author has no experience of this invaluable mode of accelerating cure in over-distended joints, and preventing subsequent weakness from stretching of ligaments, otherwise we are confident he would not pass it over as he does with a casual damnatory remark. Evaporating lotions, locally; aconite, salicylate of soda, and morphia, and a diet of "milk, soup or broth, and farinaceous foods," sum up the best of Mr. Marsh's treatment of cases of acute synovitis. On one occasion, in St. Bartholomew's, it seems that Mr. Willett incised and drained a knee-joint for hydrarthrosis, and several months afterwards the patient had a joint "possessed of considerable movement." This precious case has a whole page to itself, and there is no other. We should have thought that if incision were kept for other cases, and aspiration with elastic compression for such as this, better surgery would have been practised.

We have no space to go over the whole book in this critical spirit: in too many instances it is possible to do so. It is because in some respects we believe the book will do harm, by pushing our knowledge backwards, that we speak definitely our opinion. Where it can only do good by calling special attention to a class of diseases but little understood, and by handling their description in a clear and vigorous manner, we have only praise to bestow. We are only sorry that in showing his ability to deal with these so satisfactorily, he has by comparison made more conspicuous the defects in other portions of the work.

